# Updates on the genetic variations of Norovirus in sporadic gastroenteritis in Chungnam Korea, 2009-2010

**DOI:** 10.1186/1743-422X-9-29

**Published:** 2012-01-24

**Authors:** KwiSung Park, SangGu Yeo, HyeSook Jeong, KyoungAh Baek, DongUk Kim, MyoungHee Shin, JaeHyoung Song, SooJin Lee, YoungJin Choi, JoonSoo Park, SungChan Cho, DooSung Cheon

**Affiliations:** 1 Chungcheongnam-Do Institute of Health and Environmental Research, Daejeon, Korea; 2 Division of Enteric and Hepatitis Viruses, Center for Infectious Diseases, National Institute of Health, Korea Center for Disease Control and Prevention, 187 Osongsaengmyeong2(i)-ro, Gangoe-myeon, Cheongwon-gun, Chungcheongbuk-do, Korea; 3 Bio-Therapeutics Research Institute, Korea Research Institute of Bioscience and Biotechnology, 685-2 Yangcheong-ri, Ochang-eup, Cheongwon-gun, Chungcheongbuk-do 363-883, Korea; 4 Department of Pediatrics, College of Medicine, Eulji University, Daejeon, Korea; 5 Departments of Laboratory Medicine, College of Medicine, Soonchunhyang University, Cheonan, Korea; 6 Departments of Pediatrics, College of Medicine, Soonchunhyang University, Cheonan, Korea

## Abstract

Previously, we explored the epidemic pattern and molecular characterization of noroviruses (NoVs) isolated in Chungnam, Korea in 2008, and the present study extended these observations to 2009 and 2010. In Korea, NoVs showed the seasonal prevalence from late fall to spring, and widely detected in preschool children and peoples over 60 years of age. Epidemiological pattern of NoV was similar in 2008 and in 2010, but pattern in 2009 was affected by pandemic influenza A/H1N1 2009 virus. NoV-positive samples were subjected to sequence determination of the capsid gene region, which resolved the isolated NoVs into five GI (2, 6, 7, 9 and 10) and eleven GII genotypes (1, 2, 3, 4, 6, 7, 8, 12, 13, 16 and 17). The most prevalent genotype was GII.4 and occupied 130 out of 211 NoV isolates (61.6%). Comparison of NoV GII.4 of prevalent genotype in these periods with reference strains of the same genotype was conducted to genetic analysis by a phylogenetic tree. The NoV GII.4 strains were segregated into seven distinct genetic groups, which are supported by high bootstrap values and previously reported clusters. All Korean NoV GII.4 strains belonged to either VI cluster or VII cluster. The divergence of nucleotide sequences within VI and VII intra-clusters was > 3.9% and > 3.5%, respectively. The "Chungnam(06-117)/2010" strain which was isolated in June 2010 was a variant that did not belong to cluster VI or VII and showed 5.8-8.2%, 6.2-8.1% nucleotide divergence with cluster VI and VII, respectively.

## Background

The noroviruses (NoVs) are classified in the genus *Norovirus *within the family *Caliciviridae *and are now considered the most important cause of outbreaks and sporadic cases of non-bacterial gastroenteritis worldwide [1]. Patients who are infected by NoVs usually show gastrointestinal manifestations including diarrhea, vomiting, abdominal pain, and low grade fever, and almost all of the infected cases resolve spontaneously [2]. NoV strains exhibit wide genetic diversity, and both genogroup GI and GII and different genotypes within the genogroups cocirculate in a given geographical region at the same time [3].

NoV is a small round virion containing a single-stranded, positive-sense, 7.4-7.7 kb polyadenylated RNA genome, and 27-35 nm in diameter. The viral genome encodes three open reading frames (ORFs): ORF1, ORF2, and ORF3 [4-6]. Genetically, NoVs have been classified into five major groups: GI to GV. GI, GII, and GIV generally infect humans; GIII and GV infect bovine and murine species, respectively. GI, GII, and GIII have been subdivided into 14, 17, and 2 genetic clusters, respectively, whereas both GIV and GV have only one cluster [7,8].

Seeing the high variability of NoV seasonality, it seems to be other factors govern transmission pattern of disease except environmental factors. Immunity to NoV infection and disease is generally temporal and heterotypic protection is limited [9]. Additionally, NoVs are highly infectious. Due to these combined factors, nearly all children will have had at least once NoV infection by their fifth birthday. However infections and disease occur throughout life as immunity wanes and new antigenic types are encountered [10]. Indeed, NoVs are constantly evolving, with the most common group of viruses (GII.4) under positive selection pressure-whereby immune escaping variants are selected for [11]. New variants with antigenic changes may escape population immunity. The emergence of such variants has been shown to be associated with substantial increases in cases worldwide [12,13]. Therefore, understanding of molecular epidemiology of NoV is very important. This study was to determine the epidemiology of NoV infection and molecular characteristics of Korean NoV GII.4 isolates.

## Materials and methods

### Stool specimens

It was collected a total of 3171 stool specimens from patients with acute gastroenteritis in Chungnam, Korea from 2009 to 2010. The fecal specimens were diluted with phosphate buffered saline to 10% suspensions, and clarified by centrifugation at 8,000Χ g for 15 min.

### RNA extraction and Detection of NoV in clinical samples

The viral RNA was extracted from the faecal supernatant using Viral Nucleic Acid Prep Kit according to the manufacturer's instructions (Greenmate Biotech, Seoul, Korea). The extracted RNA was dissolved in 50 μL of nuclease-free water and stored at -80°C until use for real-time and semi-nested RT-PCR. Real-time RT PCR for NoV detection was conducted using an AccuPower Norovirus Real-Time RT-PCR Kit (Bioneer, Daejeon, Korea) in accordance with the manufacturer's instructions; the 50 μL reaction mixtures contained 10 μL of RNA and each primer at a final concentration of 0.3 μM [14]. Reactions were performed using Exicycler™ 96 (Bioneer, Daejeon, Korea) under the following conditions: initial hold at 45°C for 15 min and 95°C for 5 min, followed by 45 cycles at 95°C for 5 sec, 55°C for 5 sec, and 25°C for 1 min. A sample with threshold cycle value < 35 and a typical sigmoid curve was defined as positive.

### Nucleotide sequencing and molecular typing

In an effort to identify NoV genotypes, it was performed direct sequencing of all samples that tested positive for NoV by the real-time RT-PCR assay. For sequencing, semi-nested RT-PCR was conducted as described previously [12,15]. Products from the semi-nested PCR were purified using a QIA quick PCR purification kit (Qiagen, Hilden, Germany). The purified DNA was added to a reaction mixture containing 2 μL of BigDye Terminator reaction mix (ABI Prism Big Dye Terminator cycle sequencing kit; Perkin-Elmer/Applied Biosystems, Waltham, CA, USA) and 2 pmol each of the GI-R1M and GII-R1M primers. Sequencing reactions were subjected to an initial denaturation at 96°C for 1 min, followed by 25 cycles of 96°C for 10 sec, 50°C for 5 sec, and 60°C for 4 min in a thermal cycler (Gene Amp PCR System 2700; Perkin-Elmer/Applied Biosystems, Waltham, CA, USA). The products were purified by precipitating them with 100% cold ethanol, 3 M sodium-acetate (pH 5.8) before being loaded onto an automated analyzer (3730 XL DNA Analyzer; Perkin-Elmer/Applied Biosystems, Waltham, CA, USA). A BLAST search of GenBank sequences was conducted to determine the molecular type of each isolate. This was defined as the genotype that was scored as having the most nucleotides in common with the query sequence [16].

### Phylogenetic analysis

Nucleotide sequences of Korean 31 candidate NoV isolates were compared with the 20 reference sequences using Clustal W v. 2.1 [17]. Phylogenetic relationships among the ORF2 sequences of the virus isolates were determined using MEGA software v. 5.05. Maximum Composite Likelihood was used as the substitution method, while the neighbor-joining method was used to reconstruct the phylogenetic tree [18,19]. The reliability of the phylogenetic tree was determined by bootstrap re-sampling of 1,000 replicates.

### Nucleotide sequence accession numbers

The NoV candidate sequences were deposited in the GenBank sequence database (accession numbers JN688175 to JN688204).

## Results

### Epidemiological features and genotyping of NoVs

**Figure 1:**
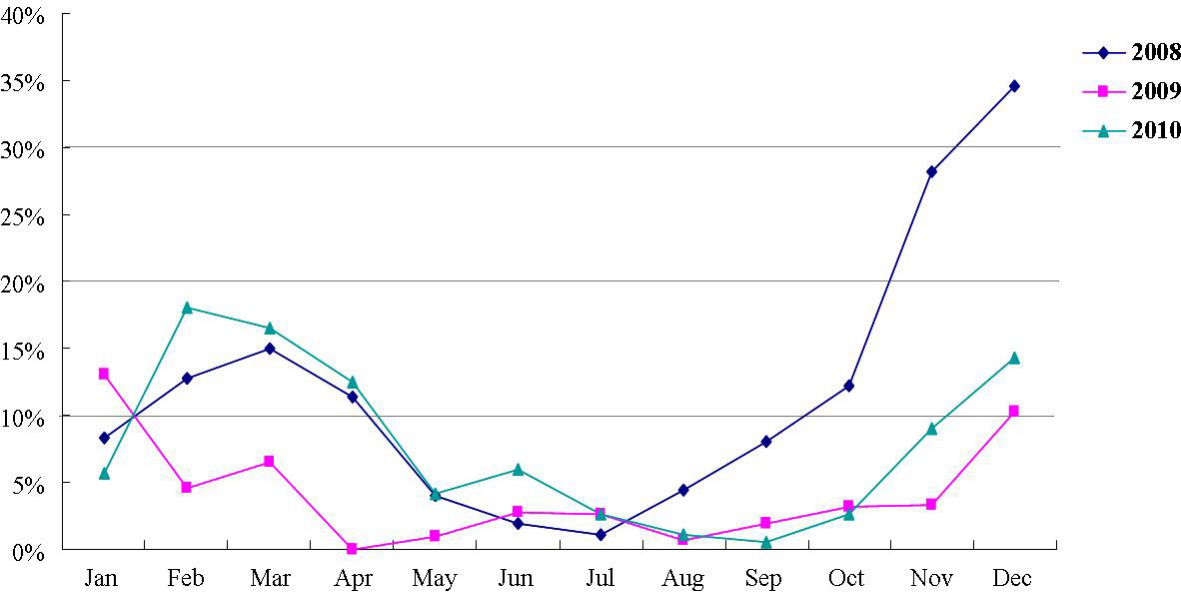
**Temporal distribution of NoV-positive cases in Chungnam, Korea, from 2008 to 2010**. The results of 2008 were reported by Park et al. [15].

We previously reported the results for epidemiological study of NoVs in Chungnam, Korea in 2008 [15]. The current study extended these findings by investigating the difference of the epidemic patterns in 2009 and 2010. In this period, 3171 samples obtained from patients with acute gastroenteritis were diagnosed for NoVs using by real-time RT-PCR and NoV positive samples were analyzed to sequences in capsid region. Consequently, it was detected a total of 211 NoVs: 64 from 1479 cases (4.3%) in 2009, and 147 from 1692 cases (8.7%) in 2010. Out of the total 211 cases, 5 cases (2.4%) were identified as GI genogroup and 206 cases (97.6%) as GII genogroup, which were further resolved into 5 GI and 11 GII genotypes, respectively. In decreasing order of abundance, these were: GII.4 (n = 130, 64.0%), GII.3 (n = 24, 11.8%), GII.8 (n = 16, 7.9%), GII.1 (n = 10, 4.9%), GII.2 and GII.7 (n = 7, 3.4%), and GII.12 and GII.16 (n = 3, 1.5%). The other thirteen genotypes (GII.6, GII.13, GII.17 and all GIs) were responsible for the remaining 1.0% of cases (Table 1). The highest occurrence of GII.4 genotype has been reported in many recent surveillance of NoV epidemic throughout world [20], which is similar to our observation with Korean NoV isolates. Temporal distribution of the NoV epidemic in Chungnam Korea was seasonal, with most cases occurred during the winter from November to April (Figure 1). The high NoV detection rate in age distribution was 19.5%, 4.7%, and 15.4% in preschool age (under 5 ages), 12.3%, 4.3%, and 8.7% in old adult group (over 60 ages), from 2008 to 2010, respectively (Table 2). The 122 were from males and 89 were from females, giving a male-to-female ratio of approximately 1.37:1.

**Table 1:** Number of NoV genotypes isolated in Chungnam, Korea, from 2009 to 2010

Genotype of NoV		2009		2010	
	
		Numbers of NoV	Percentage of subtotal	Numbers of NoV	Percentage of subtotal
GI	GI.2	-	0.0	2	0.9
	
	GI.6	-	0.0	2	0.9
	
	GI.7	-	0.0	2	0.9
	
	GI.9	-	0.0	1	0.5

	GI.10	-	0.0	1	0.5
GII	GII.1	1	1.6	9	6.1
	
	GII.2	5	7.8	2	1.4
	
	GII.3	5	7.8	19	12.9
	
	GII.4	39	60.9	91	61.9
	
	GII.6	-	0.0	1	0.7
	
	GII.7	1	1.6	6	4.1
	
	GII.8	9	14.1	7	4.8
	
	GII.12	1	1.6	2	1.4
	
	GII.13	1	1.6	-	0.0
	
	GII.16	1	1.6	2	1.4
	
	GII.17	1	1.6	-	0.0

Total		64	100	147	100

**Table 2:** Age distribution of NoV positive patients in Chungnam, Korea, from 2008 to 2010

	< 5 (Preschool age)		6 - 20 (School age)		21 - 40 (Young adult)		41 - 60 (Middle adult)		> 60 (Old adult)		Total	
	
	**Sample no**.	**Positive no**. (Percentage)	**Sample no**.	**Positive no**. (Percentage)	**Sample no**.	**Positive no**. (Percentage)	**Sample no**.	**Positive no**. (Percentage)	**Sample no**.	**Positive no**. (Percentage)	**Sample no**.	**Positive no**. (Percentage)
**2008**	687	134 (19.5%)	161	11 (6.8%)	81	4 (4.9%)	171	10 (5.8%)	368	21 (5.7%)	1,468	180 (12.3%)

**2009**	760	36 (4.7%)	135	6 (4.4%)	67	3 (4.5%)	163	8 (4.9%)	354	11 (3.1%)	1,479	64 (4.3%)

**2010**	810	125 (15.4%)	128	5 (3.9%)	110	6 (5.5%)	198	2 (1.0%)	446	9 (2.0%)	1,692	147 (8.7%)

Total	2,257	295 (13.1%)	424	22 (5.2%)	258	13 (5.0%)	532	20 (3.8%)	1,168	41 (3.5%)	4,639	391 (8.4%)

* The results of 2008 were reported by Park et al. [15]

### Phylogenetic analysis of GII.4

**Figure 2:**
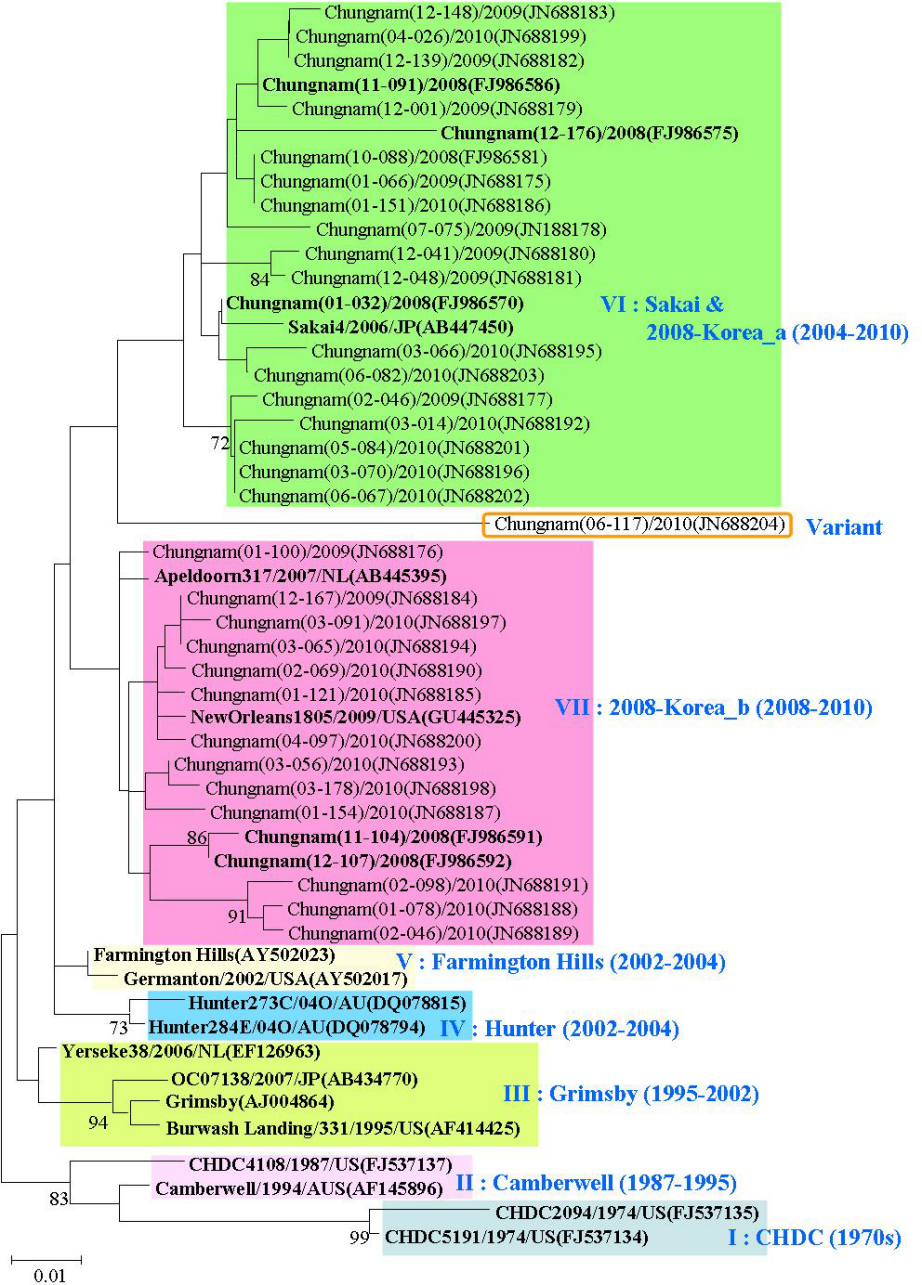
**Phylogenetic analysis based on a 237 bp sequence of the capsid region (ORF2) of NoV GII.4**. Nucleotide sequences were analyzed by neighbour joining method. The numbers at the branches indicate bootstrap values for 1,000 replicas. Reference strains were marked to bold type.

The partial nucleotide sequences of ORF2 of 30 randomly selected NoV GII.4 strains obtained from patients with acute gastroenteritis (from 2008 to 2010) were used to construct a phylogenetic tree with 20 reference strains of the same genotype extracted from GenBank database. The NoV GII.4 strains were segregated into seven distinct genetic groups, which were supported by high bootstrap values and previously reported clusters including (I) CHDC cluster (1970s)[20], (II) Camberwell cluster (1987-1995) [21], (III) Grimsby cluster (1995-2002) [22], (IV) Farmington Hills cluster (2002-2004) [2,11,23], (V) Hunter cluster (2002-2004) [24], (VI) Sakai & 2008-Korea_a cluster (2004-2008) [8,15,22], and (VII) 2008-Korea_b cluster (2008) [15]. As expected, all analyzed strains belonged to one of two previously defined Korean types of clusters, (VI) Sakai & 2008-Korea_a cluster and (VII) 2008-Korea_b cluster. The divergence of nucleotide sequence within (VI) Sakai & 2008-Korea_a intra-cluster and (VII) 2008-Korea_b intra-cluster was > 3.9% and > 3.5%, respectively. The (VI) Sakai & 2008-Korea_a cluster showed 8.6-11.7%, 5.3-7.7%, 3.5-6.3%, 3.9-5.4%, and 2.2-4.4% nucleotide divergence from cluster I to cluster V, respectively. The (VII) 2008-Korea_b cluster showed 7.7-10.6%, 4.4-6.7%, 1.7-6.2%, 2.2-5.3%, 3.0-8.1% nucleotide divergence from cluster I to VI, respectively. The "Chungnam(06-117)/2010(JN688204)" strain which isolated in June 2010 was a variant that did not belong to cluster VI and showed 5.8-8.2% nucleotide divergence from cluster VI (Figure 2).

## Discussion

Global outbreaks of acute gastroenteritis by NoVs have been frequently reported since late 1990s, and NoV has been the etiological agent in many sporadic cases of gastroenteritis in Korea. We previously reported the epidemic occurrence of NoVs in Chungnam, Korea in 2008 [15]. The current study extended these findings by investigating the difference of the epidemic pattern in 2009 and 2010. Several studies in the past have demonstrated that NoV-associated gastroenteritis occurs mainly from late fall to spring [25-28]. Overall, the results of the present study are in accordance with this general pattern, except that epidemic of NoV in Chungnam Korea was temporarily reduced in the January during 2008 to 2010. The high NoV detection rate in age distribution was 19.5%, 4.7%, and 15.4% in preschool age (under 5 ages), 12.3%, 4.3%, and 8.7% in old adult group (over 60 ages), from 2008 to 2010, respectively. Generally, the patterns of age distribution in 2008 and 2010 were similar but that of 2009 was different. The reason was considered to be aggressive hand-washing and reduction of social activities due to the pandemic H1N1 influenza in 2009.

This higher occurrence of NoV infection in young children and old people, who have weaker immunity than healthy adults, has been commonly observed. Immunity to NoV infection is temporal (between 2 and 6 months) and incomplete [9], and NoV infection frequently occurs and leads to symptoms even in adult groups unlike other viruses. In the present study, there were 5 GI and 11 GII genotypes identified. The highest prevalence of GII.4 NoV (61.6%) is consistent with recent clinical molecular epidemiological studies [20,24,29]. The NoV GII.4 strains evolved and spread in a manner similar to that of influenza A virus, with a rapid global spread of emerging variant [25]. During the last decade, most epidemics of NoV infection have been associated with the emergence of several novel GII.4 variants: CHDC, Camberwell, Grimsby, Farmington Hills, Hunter, Sakai, more recently 2008-Korea_a and b [15,20-29]. In phylogenetic and diversity analysis, all Korean isolates analyzed in this study were contained into either cluster VI or VII which had been previously defined as the representative of Korean types of NoV. The divergence of nucleotide sequences of Korean isolates within cluster VI and VII was > 3.9% and > 3.5%, respectively. The "Chungnam(06-117)/2010 (JN688204)" strain was a variant that did not belong to cluster VI or VII and showed 5.8-8.2% and 6.2-8.1% nucleotide divergence with cluster VI and VII, respectively. The RNA viruses have high mutation rate in general and new variants of GII.4 can emerge quickly [13,15,30]. In this study, it was analyzed epidemiological distribution of NoVs from acute gastroenteritis patients collected in Chungnam Korea from 2009 to 2010. It was suggested that the results of this study might reflect national trends of NoV epidemics in Korea, particularly over recent years. Molecular characterization of the Chungnam isolates also revealed patterns of variation that may be useful in future studies.

## Competing interests

The authors declare that they have no competing interests.

## Authors' contributions

KSP, SGY, HSJ, KAB, DUK, MHS, and JHS performed molecular diagnosis and sequence analysis. SJL, YJC, and JSP contributed to collection specimen and clinical diagnosis. SCC and DSC designed the study and critically revised the manuscript. All of the authors read and approved the final version of the manuscript.
